# Smear positivity in paediatric and adult tuberculosis: systematic review and meta-analysis

**DOI:** 10.1186/s12879-016-1617-9

**Published:** 2016-06-13

**Authors:** Amber Kunkel, Pia Abel zur Wiesch, Ruvandhi R. Nathavitharana, Florian M. Marx, Helen E. Jenkins, Ted Cohen

**Affiliations:** Department of Epidemiology of Microbial Diseases, Yale School of Public Health, New Haven, USA; Department of Epidemiology, Harvard School of Public Health, Boston, USA; Centre for Molecular Medicine Norway, Nordic EMBL Partnership, Oslo, Norway; Department of Pharmacy, University of Tromso, Tromso, Norway; Division of Infectious Diseases, Beth Israel Deaconess Medical Center, Boston, USA; Division of Global Health Equity, Brigham and Women’s Hospital and Harvard Medical School, Boston, USA

**Keywords:** Child, Diagnosis, Acid-fast bacilli, Sputum microscopy, Age-specific

## Abstract

**Background:**

Tuberculosis (TB) diagnosis continues to rely on sputum smear microscopy in many settings. We conducted a meta-analysis to estimate the percentage of children and adults with tuberculosis that are sputum smear positive.

**Methods:**

We searched PubMed, MEDLINE, Embase, and Global Health databases for studies that included both children and adults with all forms of active TB. The pooled percentages of children and adults with smear positive TB were estimated using the inverse variance heterogeneity model. This review was registered in the PROSPERO database under registration number CRD42015015331.

**Results:**

We identified 20 studies meeting our inclusion criteria that reported smear positivity for a total of 18,316 children and 162,574 adults from 14 countries. The pooled percentage of paediatric TB cases that were sputum smear positive was 6.8 % (95 % Confidence Interval (CI) 2.2–12.2 %), compared with 52.0 % (95 % CI 40.0–64.0 %) among adult cases. Eight studies reported data separately for children aged 0–4 and 5–14. The percentage of children aged 0–4 that were smear positive was 0.5 % (95 % CI 0.0–1.9 %), compared with 14.0 % (95 % CI 8.9–19.4 %) among children aged 5–14.

**Conclusions:**

Children, especially those aged 0–4, are much less likely to be sputum smear positive than adults. National TB programs relying on sputum smear for diagnosis are at risk of under-diagnosing and underestimating the burden of TB in children.

**Electronic supplementary material:**

The online version of this article (doi:10.1186/s12879-016-1617-9) contains supplementary material, which is available to authorized users.

## Background

National tuberculosis (TB) programs focused on diagnosis and treatment of sputum smear positive, highly infectious TB cases have historically under-estimated the burden of TB in children, who frequently present with smear negative, paucibacillary disease [1, 2]. Recent years, however, have brought increasing attention to the global burden of TB in children. Childhood TB was the focus of the World TB Day in 2012, and in 2012 the World Health Organization (WHO) published their first estimates of the global burden of childhood TB [2, 3].

Sputum smear microscopy remains the primary diagnostic tool available for bacteriologic diagnosis of TB in both children and adults in most settings [4]. Although conventional wisdom holds that children are less likely to present with smear positive TB than adults, the relative values of these proportions are not known with precision and systematic reviews of the relative percentages of children and adults with TB that are smear positive have not been conducted. We need quantitative estimates of these percentages to understand the extent to which national TB programs that prioritise treatment of smear positive TB will tend to under-serve their paediatric as well as adult populations. These estimates may also be used to more accurately inform the global burden of childhood TB. WHO estimates in 2012 assumed a similar degree of under-diagnosis and underreporting among children and adults. This approach would tend to under-estimate the burden of childhood TB if children are in fact less likely to be smear positive [3]. Jenkins et al. [5] provided alternative estimates of paediatric and adult rates of smear positivity based on values from studies in Norway and the United States [6, 7]; however, the specific values used in this analysis were subject to some debate [8, 9].

In this paper, we present a systematic review and meta-analysis of the relative percentages of children and adults with tuberculosis that are sputum smear positive. We also assess the percentage of children that are smear positive by age group (0–4 vs. 5–14). These percentages will be useful for improving estimates of the global burden of childhood TB and for helping to assess the potential value of more sensitive diagnostic methods in children and adults.

## Methods

The authors followed the Preferred Reporting Items for Systematic Reviews and Meta-Analyses (PRISMA) standards of quality for reporting systematic reviews [10]. The protocol for this study was registered in the PROSPERO database under registration number CRD42015015331 on January 12, 2015, prior to data extraction [11] and is available in the Additional file 1.

### Search strategy and selection criteria

We conducted a systematic search of MEDLINE (OvidSP 1946 to November Week 1, 2014), Embase (OvidSP 1974 to November Week 1, 2014), Global Health (OvidSP 1910 to 2014 Week 45), and PubMed online databases. All searches were conducted on November 19, 2014 and all results were limited to English. Our search terms were designed to capture studies about TB that included both children and adults and that attempted to diagnose all forms of TB. We also included search terms intended to capture the range of study designs that we thought could be used to collect data that could inform our estimates. We did not specifically include terms related to sputum smear microscopy to increase the likelihood of finding data from studies for which the sensitivity of smear microscopy was not the primary objective.

We reviewed abstracts from this initial search using a defined set of inclusion criteria. All studies that could not be excluded with certainty based on the information in the abstract were referred for full text review. Our inclusion criteria were as follows. First, all included studies needed to provide primary data on the total number of children and adults with TB, as well as the number of those children and adults with smear-positive TB. Studies should have used a cut-off age of 15 years to distinguish between children and adults, with children defined as age <15 years or, if not reported otherwise, ≤15 years. We excluded studies that did not include at least 10 cases of active TB, including at least two children and at least two adults. We also excluded studies that restricted the ages of the included children or adults (e.g. that excluded children age <5 years), did not describe an attempt to diagnose all forms of TB (i.e. pulmonary and extra-pulmonary), or required bacteriological confirmation for TB diagnosis. The latter condition was necessary as bacteriological confirmation may be unavailable for a large proportion of children with TB, reflecting the continued lack of sufficiently sensitive diagnostic tests [1]. Finally, we excluded studies that selected for participant infectiousness or other health statuses or conditions (excepting HIV) that could make the study population unrepresentative of the general population of TB cases. Our complete search strategy is included in the Additional file 1.

Two reviewers (AK and RRN) double reviewed all abstracts in EndNote X7 to rule out studies that clearly did not meet our inclusion criteria, with discrepancies mediated by a third reviewer (HEJ or TC) and uncertain studies referred for full text review. The same reviewers also double reviewed all full text articles.

### Data extraction

Two reviewers (AK and RRN) performed double data extraction and entry using Microsoft Excel. A third reviewer (HEJ or TC) arbitrated any discrepancies between the two reviewers.

From each study, we extracted the total number of children, adults, smear-positive children, and smear-positive adults with active TB. We also extracted data on study location, design, and year, as well as, when available, potential covariates including the prevalence of HIV, history of BCG, and history of previous TB among child and adult participants. The majority of these covariates were available only for a small subset of the included studies. For studies that provided a finer age breakdown than child vs. adult, we extracted the age range, total number of TB cases, and number of smear-positive TB cases from each age group.

### Quality assessment

We developed a modified QUADAS-2 tool to assess the quality of the included studies [12]. This tool is provided in the Additional file 1.

### Statistical analysis

To calculate the percentage of childhood TB cases that were smear positive, we defined the numerator as the number of children reported as smear positive and the denominator as the total number of children diagnosed with TB. Because we were interested in estimating the percentage of all childhood TB cases that were smear positive, incorporating all reasons for the absence of a smear positive result, we did not restrict our analysis to patients in whom a smear test was conducted. When smear results were clearly reported for only a subset of the study population (e.g. only among new cases), we restricted our analyses to this sub-population. We repeated this procedure among adults to calculate the percentage of adult cases that were smear positive.

We performed meta-analyses using the inverse-variance heterogeneity (IVhet) model [13], which is specifically developed for meta-analyses with high heterogeneity, in place of a random effects model, which may underestimate the width of the confidence intervals and become increasingly unreliable as heterogeneity increases. The IVhet model is implemented in the meta-analysis package MetaXL [14]. We included stratifications by age and study design in our final analyses; other potential stratification variables such as BCG and HIV were also considered.

### Notification data

In a parallel analysis, we extracted data on the total and smear-positive numbers of children and adults from the WHO TB case notification database [15]. Countries started reporting both smear positive and smear negative cases in 2006, and at the date of extraction not all data for 2013 were available and we therefore restricted our analysis to 2006–2012. We estimated the proportion of children and adults with smear-positive TB for all countries, OECD member countries, and countries with WHO benchmark status. The same statistical approach was used for the notification data as for the systematic review.

## Results

The following section presents results of our systematic review and separate analysis of WHO notification data.

### Description of included studies

**Fig. 1:**
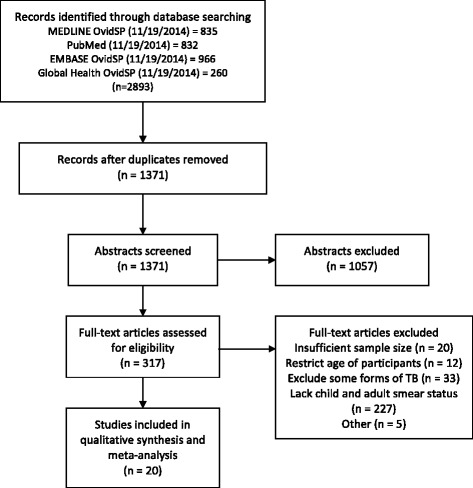
Deatils of literature search leading to the selection of included studies

The results of the literature search are displayed in Fig. 1. Our initial search yielded 1371 abstracts. Of these, 317 were referred for full text review, and 20 full texts were selected for inclusion in the qualitative synthesis and meta-analysis [16–35]. Table 1 and Additional file 2 provide information on each of the included studies. Two of the 20 included studies applied a cut-off age for children of ≤15 years [24, 28]; the remainder defined the cut-off age for children as <15 years. The 20 included studies reported smear positivity for a total of 18,316 children and 162,574 adults across 14 countries. The number of children included in each individual study ranged from six to 4691. One of the 20 studies included data on two distinct populations (adult and child index cases as well as adult and child contacts); these two populations were treated in the statistical analysis as though they were derived from two different studies [18].

**Table 1:** Summary of included studies

		Data collection		Design	Number of				Additional diagnostic methods			
Name	Location	Start year	End year	Contact tracing?	Children	Adults	Smear + children	Smear + adults	X-ray	Culture	Signs and symptoms	Histopathology
Batra	Pakistan	2008	2010	Children Only	121	1994	29	1089	Yes	Unclear	Yes	Yes
Behera	India	2002	2008	No	2549	25612	383	12984	Unclear	Unclear	Unclear	Unclear
British Thoracic Association A	United Kingdom	1973	1974	No	123	1114	30	214	Unclear	Yes	Unclear	Yes
British Thoracic Association B	United Kingdom	1973	1976	Yes	65	98	2	10	Yes	Yes	Yes	Yes
Capewell	United Kingdom	1977	1981	Yes	28	50	1	12	Yes	Yes	Unclear	Unclear
Feldacker	Malawi	2008	2010	No	338	10143	36	2704	Yes	Unclear	Yes	Yes
Getahun	Ethiopia	2004	2009	No	459	5991	58	1594	Yes	Yes	Yes	Yes
Harries 1	Malawi	1998	1998	No	2739	20243	127	9335	Yes	Unclear	Yes	Yes
Harries 2	Malawi	1986	1995	No	4691	14686	83	5609	Yes	Unclear	Yes	Yes
Henegar	Democratic Republic of Congo	2006	2007	No	830	5685	141	3736	No	No	Yes	No
Hoa	China, Cambodia, Vietnam	2003–2004	2004–2005	No	360	37272	111	24854	Unclear	Unclear	Unclear	Unclear
Jackson-Sillah	The Gambia	2002	2004	Yes	16	17	1	5	Yes	Yes	Yes	Unclear
Khazaei	Iran	1998	2002	No	163	2442	18	1329	Yes	Unclear	Yes	Yes
Lienhardt	Senegal	2004	2006	Yes	6	46	4	30	Yes	Yes	Yes	Unclear
Lopez	Angola	2009	2010	No	428	997	25	880	No	No	Yes	Unclear
Mukherjee	India	2008	2011	No	49	1182	11	809	Unclear	Unclear	Unclear	Unclear
Norval	Cambodia	1996	1996	No	150	6876	26	5923	Unclear	Unclear	Unclear	Unclear
Rama Prakasha	India	1995	2010	No	68	990	12	388	Yes	Yes	Yes	Yes
Ramos	Ethiopia	1998	2007	No	1029	1194	132	514	Yes	Unclear	Yes	Yes
Tagaro	Vanuatu	2007	2011	No	136	432	15	185	Yes	Unclear	Yes	Unclear
Wood	South Africa	2009	2009	No	3968	25510	194	12117	Unclear	Yes	Unclear	Unclear

Of the included studies, eight provided data on child TB cases separately for younger and older children (total and smear positive only), with a cut-off age of 4 years. One of these eight studies used an age cut-off for younger children of <4 years [29]; for the remaining studies, the cut-off used was ≤4 years. The eight studies included data on a total of 4922 children in the younger age group and 2587 children in the older age group.

Although we sought to extract data on several potential covariates including history of BCG vaccination, HIV infection, and previous TB treatment, most studies did not provide sufficient information to include these covariates in the analyses (Additional file 2). Data on BCG vaccination was provided by only one of the 20 included studies, and HIV infection by six (including two with high rates of missing data).

Quality assessments of the included studies are provided in the Additional file 1. The included studies could be broadly classified as either contact tracing studies or retrospective record-based studies. We assessed contact tracing studies as having a study population possibly not representative of the general population of TB cases; however, these studies often included extensive attempts to accurately diagnose TB among children (Table 1). In contrast, we assessed the majority of the record-based studies as being sufficiently representative of the general population of TB cases, but given the more limited range of tests used to diagnose TB, these studies may have diagnosed TB in children with less reliability (Table 1).

### Pooled percentage smear positive: children vs. adults

**Fig. 2:**
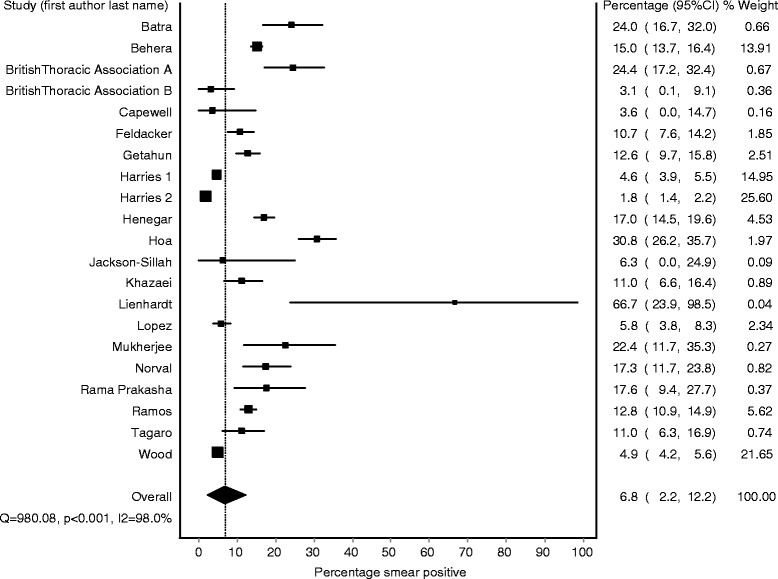
Forest plot of smear positivity percentage among children

**Fig. 3:**
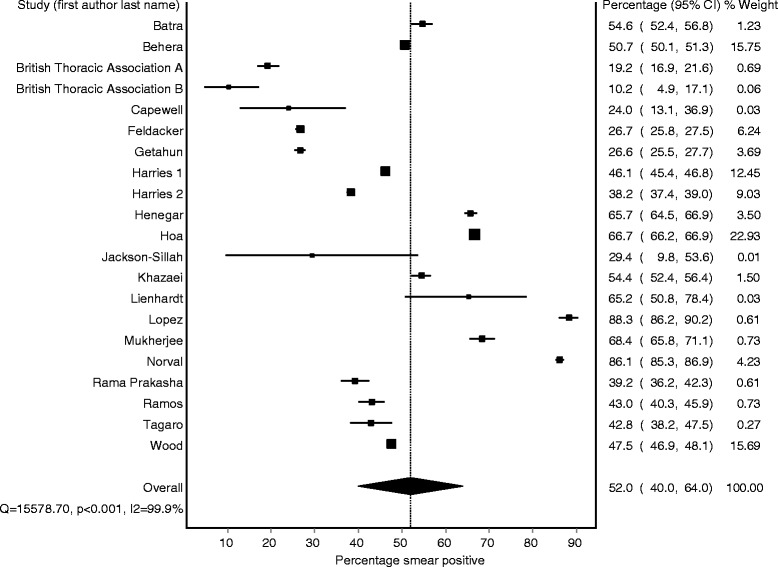
Forest plot of smear positivity percentage among adults

Figures 2 and 3 show the smear positivity percentage among children and adults respectively in the 20 included papers. Results stratified by study design (contact tracing vs record-based) are included in the Additional file 1. We found a high degree of heterogeneity in the results across the included studies that could not be explained by differences in design (I^2^ ≥ 98 % for both adults and children). Using the IVhet method to account for this heterogeneity [13], we obtained a pooled estimate for the percentage of TB cases with smear positive disease of 6.8 % (95 % CI 2.2–12.2 %) among children and 52.0 % (95 % CI 40.0–64.0 %) among adults.

### Pooled percentage smear positive: younger children vs. older children

**Fig. 4:**
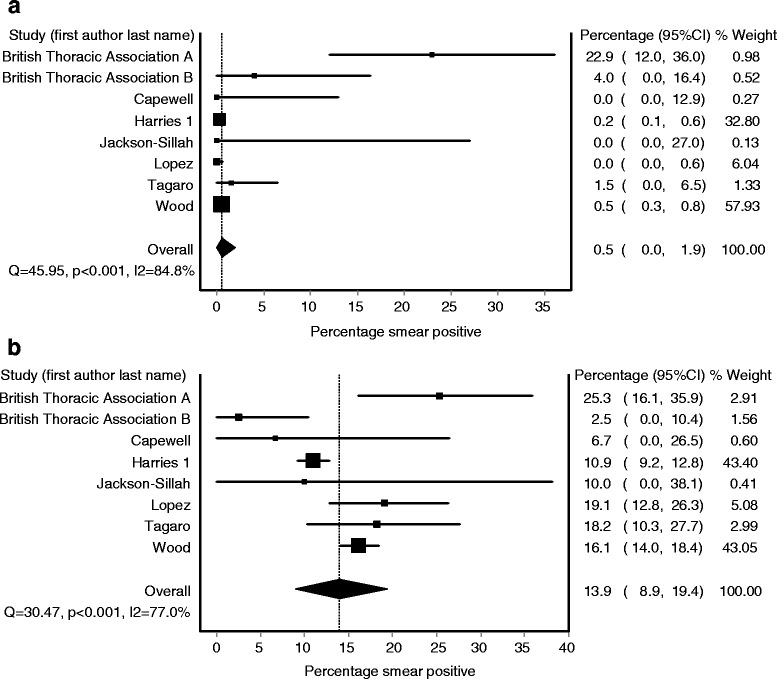
Forest plots of smear positivity percentage among (**a**) younger children (ages 0-4 years) and (**b**) older children (ages 5-14 years)

Figure 4 depicts the smear positivity of younger and older children respectively in the eight studies that provided data broken down into these two age groups (with a cut-off age of 4 years). These results also display considerable heterogeneity, though less so than the measures for children vs. adults (I^2^ = 85 % for younger children and 77 % for older children). Using the IVhet method to account for this heterogeneity [13], we obtained a pooled estimate for the percentage of TB cases with smear positive disease of 0.5 % (95 % CI 0.0–1.9 %) among younger children aged 0–4 and 14.0 % (95 % CI 8.9–19.4 %) among older children aged 5–14.

### Analysis of notification data

The results of our parallel analysis of WHO notification data are included in the Additional file 1. Our examination of these data revealed a number of limitations, including an unexpectedly high numbers of countries reporting either 0 % or 100 % of cases to be smear positive; the high frequency of cases reported as “smear unknown”; and the high variability in the proportion of cases reported as “smear unknown” even among benchmark and OECD countries. Because of these limitations, these data were not included in our primary analysis. Although the age specific estimates of smear positivity derived from these notification data vary by which countries are included in the analysis (see Additional file 1 for details), the overall results agree with our qualitative assessment of substantially lower rates of smear positivity among children than among adults.

## Discussion

In this study, we conducted a systematic review and meta-analysis to assess the relative percentages of paediatric and adult TB cases that present with sputum smear positive TB. We estimated that just 6.8 % of paediatric TB cases are sputum smear positive, compared with 52.0 % of adults. Furthermore, we show that this percentage varies greatly depending on the age of the children, with the percentage smear positive just 0.5 % among children aged 0–4 compared with 14.0 % among children aged 5–14. Our results are consistent with the assumptions of Jenkins et al. [5], who assumed a smear positivity percentage of 8.8 % among childhood TB cases compared with 49.5–56.6 % among adults when accounting for the under-diagnosis of children to estimate the global burden of childhood TB.

We found a high degree of heterogeneity across studies. The heterogeneity was reduced when we stratified children into age groups of 0–4 and 5–14 years, suggesting that differences in the age distributions across studies may explain some of this variability. The age structure of the included study populations was highly heterogeneous, with the percentage of children age <5 years ranging from 38 % [18] to 80 % [35] in the individual studies for which these data were available. However, fewer than half of the included studies provided data on the age distribution of the included children. The overall percentage of children with smear positive TB is likely to vary depending on the age structure of the population of interest, as well as the average age of infection. The percentage of paediatric TB cases who are smear positive may also differ across settings based on other factors such as the prevalence of HIV, the prevalence of BCG vaccination, and the average duration of disease prior to diagnosis. We were not able to assess many of these factors in this meta-analysis given the limited number of studies meeting the inclusion criteria.

In defining the inclusion criteria, we were careful to exclude studies that were clearly biased towards or against the mostly highly infectious TB cases (e.g. record-based studies from hospitals in which smear-positive patients were routinely hospitalised; contact tracing studies that reported data only on those adults with at least one infected child contact). However, the included study designs were not without limitations. Cases discovered through contact tracing may be less severe and less likely to be smear positive than those identified through routine surveillance. On the other hand, routine diagnosis of childhood TB, particularly in low-resource settings, is often heavily reliant on clinical diagnosis and thus may not represent the true burden of disease. Though we extracted data on diagnosis patterns from each study (Table 1), it was often difficult to assess the degree to which diagnostic aggressiveness and willingness to rely on clinical diagnosis differed across the included studies. Concerns about the potential effects of study design on representativeness and over- or under-diagnosis of TB cases are reflected in our quality assessments of the included studies provided in the Additional file 1.

New tools and methods to diagnose TB in children are needed given the low sensitivity of sputum smear microscopy and lack of other inexpensive and reliable methods in this highly vulnerable population. Recent studies have addressed the potential role of nucleic acid amplification tests such as Xpert MTB/RIF in diagnosing paediatric TB [36, 37]. The sensitivity of Xpert MTB/RIF among children appears to exceed that of smear microscopy; however, the sensitivity is still low among culture-negative presumptive TB cases, which account for a large fraction of the burden of TB among children [1, 36, 37].

## Conclusions

The low percentages of smear positivity in this analysis emphasise the degree to which national TB programs dependent on sputum smear microscopy will need to rely on clinical diagnoses of TB in children. Use of Xpert MTB/RIF would likely reduce but not eliminate this problem. Attempts to estimate the burden of TB in children locally or globally should account for the relative degree of under-diagnosis and underreporting caused by the limitations of existing methods for diagnosing childhood TB.

## Abbreviations

IVhet, inverse-variance heterogeneity; TB, tuberculosis; WHO, World Health Organization

## Additional file

Additional file 1 Supplemental information for A: Search strategies, B: Rational for full text exclusions, C: Study quality assessment tool, D: Study quality assessment results, E: Stratified results by age group, F: Stratified results by study design, G: Challenges of using notification data, H: Analysis of notification data. (DOCX 612 kb) Additional file 2 Table of additional details for included studies. (XLSX 52 kb)
