# Supplementary material for: Smear positivity in paediatric and adult tuberculosis: systematic review and meta-analysis
Source: BMC Infect Dis. 2016 Jun 13;16:282. doi: 10.1186/s12879-016-1617-9 (PMC4906576; doi:10.1186/s12879-016-1617-9)
Supplement: Additional file 1 — Supplemental information for A: Search strategies, B: Rational for full text exclusions, C: Study quality assessment tool, D: Study quality assessment results, E: Stratified results by age group, F: Stratified results by study design, G: Challenges of using notification data, H: Analysis of notification data. (DOCX 612 kb) [file 12879_2016_1617_MOESM1_ESM.docx]

**A. Search strategies**

Medline (OvidSP), Embase (OvidSP), and Global Health (OvidSP):

| **#** | **Search** |
| --- | --- |
| 1 | Exp Tuberculosis |
| 2 | (tuberculosis or tb).ti. |
| 3 | 1 or 2 |
| 4 | Exp Infant/ |
| 5 | Exp Chid/ |
| 6 | (child* or infant* or neonate* or newborn* or new born* or baby or babies or toddler* or boy* or girl* or pediatric* or paediatric* or prepubescen* or prepuberty).tw. |
| 7 | 4 or 5 or 6 |
| 8 | Exp Adult/ |
| 9 | adult*.tw. |
| 10 | 8 or 9 |
| 11 | Exp Follow-Up Studies/ |
| 12 | Exp Retrospective Studies/ |
| 13 | Exp Prospective Studies/ |
| 14 | Exp Contact Tracing/ |
| 15 | Exp Mass Screening/ |
| 16 | Exp Disease Notification/ |
| 17 | (retrospective or routine or regist* or screening or contact* or case finding* or mass screening or population based or cohort* or source case* or outbreak* or index case* or burden or incidence or prevalence or household* or notification*).tw. |
| 18 | 11 or 12 or 13 or 14 or 15 or 16 or 17 |
| 19 | (extra pulmonary or extrapulmonary or extra PTB or EPTB or clinical or radiologic or radiograph or radiography or x ray or chest radiograph or chest x ray or probable or forms or types or symptom* or sign or signs or empiric* or disseminated or confirm* or presumed).tw. |
| 20 | (smear or sputum or notify* or active or types or forms or regist*).tw. |
| 21 | 3 and 7 and 10 and 18 and 19 and 20 |
| 22 | Limit to English language |

Pubmed

| **#** | **Search** |
| --- | --- |
| 1 | "Tuberculosis"[ MeSH] OR "tuberculosis"[title] OR TB[title] |
| 2 | Infant[MeSH Terms] OR Child[MeSH Terms] OR child*[tiab] OR infan*[tiab] OR neonate*[tiab] OR newborn*[tiab] OR new born*[tiab] OR baby[tiab] OR babies[tiab] OR toddler*[tiab] OR boy[tiab] OR boys[tiab] OR girl*[tiab] OR pediatric[tiab] OR "paediatric"[tiab] OR prepubescen*[tiab] OR prepuberty*[tiab] |
| 3 | adult[mesh] OR adult*[tiab] |
| 4 | follow-up studies[MeSH] OR retrospective studies[mesh] OR prospective studies[MeSH] OR contact tracing[MeSH] OR mass screening[MeSH] OR disease notification[MeSH] OR retrospective[tiab] OR routine[tiab] OR regist*[tiab] OR screening[tiab] OR contact*[tiab] OR case finding[tiab] OR case-finding[tiab] OR mass screening[tiab] OR population-based[tiab] OR cohort*[tiab] OR source case* [tiab] OR outbreak* [tiab] OR index case*[tiab] OR burden[tiab] OR incidence[tiab] OR prevalence[tiab] OR household*[tiab] OR notification*[tiab] |
| 5 | extra pulmonary OR extrapulmonary[tiab] OR extra-pulmonary[tiab] OR extra-PTB[tiab] OR EPTB[tiab] OR clinical[tiab] OR radiologic[tiab] OR radiograph[tiab] OR radiography[tiab] OR x-ray[tiab] OR chest radiograph[tiab] OR chest X-ray[tiab] OR probable[tiab] OR forms[tiab] OR types[tiab] OR symptom*[tiab] OR sign[tiab] OR signs[tiab] OR empiric*[tiab] OR disseminated[tiab] OR confirm*[tiab] OR presumed[tiab] |
| 6 | smear[tiab] OR sputum[tiab] OR notifi*[tiab] OR active[tiab] OR types[tiab] OR forms[tiab] OR regist*[tiab] |
| 7 | 1 AND 2 AND 3 AND 4 AND 5 AND 6 |

Overview of PubMed terms

1: Captures studies primarily about TB

2: Including child patients

3: As well as adult patients

4: Words indicating study designs that could be used to capture relevant data

5: Words indicating an attempt to extra-pulmonary TB and/or lack of dependence on bacteriological diagnosis (an attempt to diagnose all forms of TB)

6: Words indicating studies that included a broad spectrum of TB types (an attempt to diagnose all forms of TB). These terms were chosen based on their empirical ability to improve the relevance of the search results.

**B. Specific reasons for full text exclusions**

This includes additional information about the studies that were excluded after full-text review. Studies falling into more than one category were assigned to the first category (top to bottom) that applied.

Insufficient sample size

Not ≥10 active TB cases (≥2 children and ≥2 adults): 20

Restrict age of participants

Age restrictions on included participants: 12

Exclude some forms of TB

Specific TB type (any but pulmonary): 3

Specific TB type (pulmonary only): 21

Only bacteriological confirmation: 7

Select for infectiousness: 2

Lack child and adult smear status

Lack age-disaggregated smear status: 225

Different age cut-offs used for children and adults: 2

Other

Duplicate study population: 2

Full text could not be located: 1

Could not determine which numbers to extract: 2

**C. Modified QUADAS 2 tool used to assess study quality**

We used a modified version of the QUADAS 2 tool to assess the quality of the included studies. The QUADAS 2 tool is intended to assess the quality of diagnostic studies; a few modifications were necessary to address the deviations of this review from standard assessments of diagnostic quality (e.g. the lack of a clear reference standard). Quality assessments were completed by AK and reviewed by TC. The overall risk of bias for each section was assessed as “high” if our responses to any of the signalling questions showed signs of potential bias, and “unclear” if any questions were unable to be answered based on the provided information.

Patient Selection

*Describe method of patient selection:*

*Was a consecutive or random sample of patients enrolled? Yes No Unclear*

*Did the study avoid inappropriate exclusions? Yes No Unclear*

*Could the study have biased towards or against highly infectious cases? If so, describe why. Yes No Unclear*

*Overall risk of bias in patient selection: Low High Unclear*

Index Test (Smear Microscopy)

*Describe the index test and how it was conducted and interpreted:*

*Could the conduct or interpretation of the index test have introduced bias? Justify answer. Low High Unclear*

Additional Diagnostic Methods

*Describe the other diagnostic procedures used to diagnose TB.*

*Sensitivity of diagnostic procedure: Is the diagnostic procedure likely to capture all forms of TB, including those for which bacteriological confirmation may not be obtained? If so, describe why. Yes No Unclear*

*Specificity of diagnostic procedure: Is the diagnostic procedure likely to lead to overdiagnosis of TB? If so, describe why. Yes No Unclear*

*Could the diagnostic procedure, its conduct, or its interpretation have introduced bias? Low High Unclear*

**D. Results of modified QUADAS 2 quality assessment.**

| Study | Risk of Bias | | |  |
| --- | --- | --- | --- | --- |
| (First author last name) | Patient Selection | Index Test | Additional Diagnostic Methods |  |
| Batra | H | ? | ? |  |
| Behera | L | ? | ? |  |
| British Thoracic Association A | L | ? | ? |  |
| British Thoracic Association B | H | ? | ? |  |
| Capewell | H | ? | ? |  |
| Feldacker | L | ? | H |  |
| Getahun | L | ? | H |  |
| Harries 1 | L | ? | H |  |
| Harries 2 | L | H | H |  |
| Henegar | L | ? | H |  |
| Hoa | L | ? | ? |  |
| Jackson-Sillah | H | ? | L |  |
| Khazaei | L | ? | H |  |
| Lienhardt | H | ? | L |  |
| Lopez | L | L | H |  |
| Mukherjee | L | ? | H |  |
| Norval | ? | L | ? |  |
| Rama Prakasha | L | ? | H |  |
| Ramos | L | ? | H |  |
| Tagaro | L | ? | ? |  |
| Wood | L | ? | ? |  |
|  |  |  |  |  |
| L: low, H: high, ?: unclear |  |  |  |  |

**E. Results by age group: adults**

We were able to stratify 9 populations across 8 studies into four adult age groups (15-24, 25-44, 45-64, and 65+) (1-8). The percentage smear positive did not vary substantially across these age groups.

Age 15-24: 56.4% (95% CI: 36.5%, 75.8%), I2=99.3%

Age 25-44: 54.6% (95% CI: 29.6%, 79.0%), I2=99.8%

Age 45-64: 63.4% (95% CI: 36.9%, 87.9%), I2=99.7%

Age 65 plus: 62.5% (95% CI: 30.5%, 91.6%), I2=98.7%

**F. Results stratified by study design**

We calculated the percentage of children and adults that were smear positive stratified by study design (contact tracing or not).

In five studies the child participants were identified through contact tracing (4, 7-10). Among the contact tracing studies, the pooled percentage of children that were smear positive was 14.2% (95% CI: 0.2% - 33.9%), I2=86.1%. For the remaining studies, the percentage of children that were smear positive was 6.7% (95% CI: 2.2% - 12.1%), I2=98.4%.

In four studies the adult participants were identified through contact tracing (4, 7, 8, 10). Among the contact tracing studies, the pooled percentage of adults that were smear positive was 25.3% (95% CI: 1.1%, 56.3%), I2=93.5%. For the remaining studies, the percentage of children that were smear positive was 52.1% (95% CI: 40.1%, 64.0%), I2=99.9%.

In both age groups, the number and sizes of studies performing contact tracing were small, leading to large confidence intervals around these estimates.

**G. Challenges of using notification data to estimate smear positivity**

We identified a number of challenges involved in using notification data to estimate the percentage of children and adults with TB who are smear positive.

1. A highly disproportionate number of countries report that either 0% or 100% of adults or children with TB are smear positive, as shown in the figure below. Data are shown for all countries that reported data to the WHO between the years 2006 and 2012 inclusive.

2. OECD and Benchmark countries report a high proportion of pulmonary TB cases with unknown smear positivity status, reflecting both the reliance on other diagnostic methods and inconsistent reporting. These data are also taken from WHO notification data from 2006-2012 and focus on the subset of countries that form the OECD countries and a group identified by the WHO to have particularly high quality TB surveillance (“Benchmark countries^[[1]](#footnote-1)^”).

3. Even within the category of benchmark and OECD countries, the proportion of cases with unknown smear status varies greatly, further representing the unreliability of these data. These data are again taken from WHO notification data, 2006-2012, as above.

**H. Analysis of notification data**

The following plots show the results of our analysis of the notification data. For the analyses of data on children, we excluded countries reporting 0% or 100% of children as smear-positive. The same restrictions were applied to data on smear-positive TB in adults..

*Notification data from benchmark countries - children*

*Notification data from benchmark countries - adults*

*Notification data by country category - children*

*Notification data by country category - adults*

**Supplemental References**

1. Getahun B, Ameni G, Medhin G, Biadgilign S. Treatment outcome of tuberculosis patients under directly observed treatment in Addis Ababa, Ethiopia. Braz J Infect Dis. 2013;17(5):521-8.

2. Wood R, Lawn SD, Caldwell J, Kaplan R, Middelkoop K, Bekker LG. Burden of new and recurrent tuberculosis in a major South African city stratified by age and HIV-status. PloS one. 2011;6(10):e25098.

3. Hoa NB, Wei C, Sokun C, Lauritsen JM, Rieder HL. Characteristics of tuberculosis patients at intake in Cambodia, two provinces in China, and Viet Nam. BMC Public Health. 2011;11:367.

4. Jackson-Sillah D, Hill PC, Fox A, Brookes RH, Donkor SA, Lugos MD, et al. Screening for tuberculosis among 2381 household contacts of sputum-smear-positive cases in The Gambia. Trans R Soc Trop Med Hyg. 2007;101(6):594-601.

5. Khazaei HA, Rezaei N, Bagheri GR, Dankoub MA, Shahryari K, Tahai A, et al. Epidemiology of tuberculosis in the Southeastern Iran. Eur J Epidemiol. 2005;20(10):879-83.

6. Norval PY, San KK, Bakhim T, Rith DN, Ahn DI, Blanc L. DOTS in Cambodia. Directly observed treatment with short-course chemotherapy. Int J Tuberc Lung Dis. 1998;2(1):44-51.

7. Capewell S, Leitch AG. The value of contact procedures for tuberculosis in Edinburgh. Br J Dis Chest. 1984;78(4):317-29.

8. A study of a standardised contact procedure in tuberculosis. Report by the Contact Study Sub-Committee of The Research Committee of the British Thoracic Association. Tubercle. 1978;59(4):245-59.

9. Batra S, Ayaz A, Murtaza A, Ahmad S, Hasan R, Pfau R. Childhood tuberculosis in household contacts of newly diagnosed TB patients. PloS one. 2012;7(7):e40880.

10. Lienhardt C, Fielding K, Hane AA, Niang A, Ndao CT, Karam F, et al. Evaluation of the prognostic value of IFN-gamma release assay and tuberculin skin test in household contacts of infectious tuberculosis cases in Senegal. PloS one. 2010;5(5):e10508.

1. Benchmark countries include: Australia, Canada, Denmark, Germany, Japan, the Netherlands, New Zealand, Sweden, the United Kingdom, and the United States of America [↑](#footnote-ref-1)
